# Treatment planning with a 2.5 MV photon beam for radiation therapy

**DOI:** 10.1002/acm2.13811

**Published:** 2022-10-27

**Authors:** Navid Khaledi, Chris Hayes, Louise Belshaw, Mark Grattan, Rao Khan, James L. Gräfe

**Affiliations:** ^1^ Department of Physics Faculty of Science Toronto Metropolitan University Toronto Ontario Canada; ^2^ Radiotherapy Physics Northern Ireland Cancer Centre Belfast Health and Social Care Trust Belfast UK; ^3^ Department of Physics and Astronomy and Department of Radiation Oncology Howard University Washington District of Columbia USA

**Keywords:** 2.5 MV photons, commissioning, IMRT, Monte Carlo simulation, radiotherapy, treatment planning

## Abstract

**Purpose:**

The shallow depth of maximum dose and higher dose fall‐off gradient of a 2.5 MV beam along the central axis that is available for imaging on linear accelerators is investigated for treatment of shallow tumors and sparing the organs at risk (OARs) beyond it. In addition, the 2.5 MV beam has an energy bridging the gap between kilo‐voltage (kV) and mega‐voltage (MV) beams for applications of dose enhancement with high atomic number (*Z*) nanoparticles.

**Methods:**

We have commissioned and utilized a MATLAB‐based, open‐source treatment planning software (TPS), matRad, for intensity‐modulated radiation therapy (IMRT) dose calculations. Treatment plans for prostate, liver, and head and neck (H&N), nasal cavity, two orbit cases, and glioblastoma multiforme (GBM) were performed and compared to a conventional 6 MV beam. Additional Monte Carlo calculations were also used for benchmarking the central axis dose.

**Results:**

Both beams had similar planning target volume (PTV) dose coverage for all cases. However, the 2.5 MV beam deposited 6%–19% less integral doses to the nasal cavity, orbit, and GBM cases than 6 MV photons. The mean dose to the heart in the liver plan was 10.5% lower for 2.5 MV beam. The difference between the doses to OARs of H&N for two beams was under 3%. Brain mean dose, brainstem, and optic chiasm max doses were, respectively, 7.5%–14.9%, 2.2%–8.1%, and 2.5%–19.0% lower for the 2.5 MV beam in the nasal cavity, orbit, and GBM plans.

**Conclusions:**

This study demonstrates that the 2.5 MV beam can produce clinically relevant treatment plans, motivating future efforts for design of single‐energy LINACs. Such a machine will be capable of producing beams at this energy beneficial for low‐ and middle‐income countries, and investigations on dose enhancement from high‐*Z* nanoparticles.

## 1 INTRODUCTION

The main goal of radiotherapy is to deliver the prescribed dose to the tumor while sparing normal tissues. To this end, different energy photon, ions, and electron beams continue to be employed in various combinations and arrangements, as well as with dose enhancers,^1^, ^2^, ^3^, ^4^, ^5^ all in the pursuit of a higher therapeutic ratio. Therefore, development of new approaches and modalities is an evolutionary process. Moreover, as the different types of radiations have different physical and biological effects and characteristics, depending on the type and site of tumor, electrons, photons, ions, or a combination of them may be used, such as mixing photons and electrons,^6^, ^7^, ^8^ photons and protons,^9^ as well as low‐ and high‐energy photons.^10^

Photon beams in the kilo‐voltage (kV) and mega‐voltage (MV) range are employed for imaging and treating cancer patients. kV x‐rays have been used for decades for superficial and orthovoltage x‐ray therapy.^11^, ^12^ Nowadays, they are mostly used for imaging and superficial therapy. In comparison to other beams, such as electrons, they are suitable for treatment of small superficial lesions due to their sharper lateral dose fall‐off and less penumbra. In contrast, the dose to bone for low‐energy photons is an issue that is caused by the photoelectric effect.^13^, ^14^ In addition, the maximum dose is at or near the surface for kV x‐rays, which limits the quality of treatment for deeper tumors. Therefore, MV photons with lower probability of photoelectric effect and higher penetration depth are better choices for treatment of nonsuperficial cases. The minimum MV range photon that is commonly used in most clinics is 6 MV. However, 6 MV and lower MV x‐rays, that is, 4 and 2.5 MV, are also utilized for inline MV imaging on linear accelerators (LINACs). The feasibility of using low‐MV photons (≤6 MV), around 4 MV, for imaging was investigated by Faddegon et al.^15^ The superiority of carbon target utilizing low‐energy electrons for production of MV imaging beam over conventional 6 MV beam was demonstrated. The resolution and contrast‐to‐noise ratio (CNR) were improved by factors of 2 and 3, respectively.

In 2012, another study^16^ was conducted for energies below 2.4 MV. They employed aluminum and carbon targets irradiated by 1.9 and 2.35 MeV electrons. However, the bremsstrahlung spectrum by carbon target contained more low‐energy photons, resulting in higher probability of photoelectric effect. This arrangement achieved a maximum 7.4 times better CNR compared to 6 MV beam. Furthermore, a recent investigation^17^ simulated a 2.5 MV beam through the Geant4 Monte Carlo (MC) code, and demonstrated that the Varian Clinac (Siemens Healthineers, Erlangen, Germany) with carbon target produces more photoelectric range photons in comparison to the standard copper target currently used on the Varian TrueBeam. The study also demonstrated a sharp dose fall‐off for carbon target percentage depth dose (PDD) measured in water. Other studies^18^, ^19^, ^20^ have been performed for characterizing the physical properties of the low‐energy photons such as PDDs, dose profiles, and energy spectrum for 2.5 MV from a TrueBeam machine. Some of them^19^, ^20^ evaluated the dose to patient of the 2.5 MV imaging photons through commissioning and modeling of the beam. The dose‐volume histograms (DVHs) of the imaging fields demonstrated that the maximum doses of 2.5 MV beam to organs at risk (OARs) can be reduced to about half of 6 MV photons.

Although several investigations^21^, ^22^, ^23^ have been conducted on low‐MV photons, to our knowledge, no treatment planning studies have been performed on 2.5 MV photons of Varian TrueBeam. The AAPM TG‐180 report^24^ has evaluated the doses to OARs for kV, 2.5 MV, and 6 MV imaging photons through model‐based treatment planning system (TPS). They indicate that the dose to bony tissues by kV imaging x‐rays can be underestimated by up to 300% by conventional TPSs due to the photoelectric effect.

The inline MV imaging beam may be better than kV beam for imaging when there are metal implants, but otherwise kV is preferred when available, because the MV beam delivers higher overall dose to patients.^20^, ^24^ In addition, all previous literature studies investigated the 2.5 MV beam for assessing doses resulting from imaging procedures. The purpose of this work is to evaluate feasibility of 2.5 MV beam for treatment planning.^25^ This was done using an open‐source planning system for six different body sites with different treatment depths.

In this work, we investigated the potential of treatment with the 2.5 MV photon beam that is an option for MV imaging on Varian TrueBeam linear accelerators. The beam is produced by bombarding 2.5 MeV electrons on a low atomic number (*Z*) copper target, and does not use any flattening filter. In terms of physical properties, the depth of maximum dose (*d*_max_) is lower than the 6 MV beam (0.6 cm vs. 1.5 cm), which is the most common beam in radiation therapy. Due to the faster dose fall‐off of the 2.5 MV beam, it can be efficient for treatment of shallow tumors and sparing the deep‐seated OARs due to the lower exit dose. In addition to being effective in treating semi‐deep tumors, as will be discussed, this beam can be produced in compact LINACs at a lower cost as an alternative to cobalt‐60 machines in developing countries.

Furthermore, as the effectiveness of high‐*Z* nanoparticles in reducing the cell survival fraction for the kV‐range photons, due to the photoelectric and Auger electrons effect, has been demonstrated previously,^3^, ^4^ the 2.5 MV beam may be considered as a suitable beam for this application. The 2.5 MV spectrum contains a greater proportion of low‐energy x‐rays peaking at around 120 keV,^17^, ^19^ and the dose enhancement with 2.5 MV photons with the presence of gold nanoparticles (GNPs) has been indicated recently in an in vitro study,^1^ while the dose enhancement for 6 MV energy is very small.^26^, ^27^ Additionally, a MC study^28^ showed that the dose enhancement due to GNPs for a 2.5 MV low atomic number target is twofold of 6.5 MV beam. Therefore, the 2.5 MV beam can be thought of as a bridging energy between the kV and conventional MV photon energies with specific advantages over 6 MV beams in terms of potential dose enhancement for high‐*Z* nanoparticles and gains over kV beams in terms of depth of penetration.

## 2 MATERIALS AND METHODS

### 2.1 2.5 MV beam data

The PDDs, dose profiles, and output factors (OF) of the 2.5 MV beam of a Varian TrueBeam (Siemens Healthineers, Erlangen, Germany) were measured for field sizes with 4, 6, 8, 10, 20, and 40 cm widths at a source‐to‐surface distance (SSD) of 100 cm, in a 50 × 50 × 50 cm^3^ PTW MP3 (PTW, Freiburg, Germany) water phantom by a PTW Semiflex ion chamber type 31010. The measured PDDs were converted to tissue maximum ratio (TMR) using a PTW MEPHYSTO Table Generator tool. The peak scatter factor (PSF) was assumed to be unity in conversion. These data (OF, TMR, and dose profiles) are required for beam modeling on matRad planning software (version 2.10.1).^29^ matRad is a MATLAB‐based open‐source planning software for particle therapy and photon intensity‐modulated radiotherapy (IMRT) inverse planning. matRad requires an intensity profile, which is a fluence curve at the isocenter for the largest field size in the diagonal direction that is converted to dose profile for each field size by matRad. This diagonal curve was obtained by extending the 40 × 40 cm^2^ crossplane dose profile in the off‐axis directions at the depth of 0.6 cm, and normalizing the dose values to unity. Then, its normalized dose values were changed several times to achieve a minimum deviation between the measured and matRad calculated dose profiles for different fields.

### 2.2 matRad data entry

For importing the measured data to matRad and generating the 2.5 MV beam database, which is a “.mat” file containing the OF, TMRs, and dose profiles, a MATLAB (version 2019b, MathWorks Inc., Natick, MA, USA) script was developed based on a matRad framework named “photonPencilBeamKernelCalc”. Through this framework, pencil beam dose convolution kernels were adjusted to fit the measured TMRs, intensity profile, and OFs data, resulting in a beam database file for the 2.5 MV beam. After obtaining the machine file, PDDs and dose profiles at the depths of 0.6, 5, 10, and 20 cm for the mentioned field sizes in a 50 × 50 × 50 cm^3^ water phantom were calculated through our in‐house MATLAB code customized for the 2.5 MV beam. This code is a modified matRad's main script (matRad.m) that reads the beam database, and consists of information about the source model, distance to the phantom surface, phantom geometry and Hounsfield units (HU), dose scoring directions and depths for obtaining the PDDs and dose profiles to compare matRad's calculated PDDs and dose profiles against the measurements. The difference between the calculated and measured PDDs and dose profiles, in 2 mm steps, was obtained to verify the accuracy of calculations. The surface dose of calculated PDDs by matRad was benchmarked against the values obtained through film dosimetry results of previously published work from our lab.^18^ In addition, global 2%/2 mm gamma (*γ*) index analysis^30^ (without a minimum dose threshold) was performed.

### 2.3 Treatment planning

Treatment plans for prostate, liver, head and neck (H&N), nasal cavity, glioblastoma multiforme (GBM), and two orbit cases were generated. The dose constraints/objectives of OARs and treatment targets, presented in Table 1, for step‐and‐shoot IMRT dose optimization were implemented according to the Quantitative Analyses of Normal Tissue Effects in the Clinic (QUANTEC),^31^ Radiation Therapy Oncology Group (RTOG), and literature recommendations. Moreover, all 2.5 MV treatment plans were compared with^29^ 6 MV beam in matRad in terms of DVH and dose differences. The beam angles were selected based on the clinical experience and tuning the angles and number of beams, similar to the clinical practice. The dose grid size was 3 mm for all cases. The ranges of segments are also listed in Table 2.

**TABLE 1:** Field arrangements and dose objective/constraints of the plans

	**Number of beams**	**Prescribed dose**	**Dose objective/constraints**
Prostate	9 Coplanar fields	68 Gy/34 fx^53^	Prescribed dose covers at least 98% of the PTV Maximum dose to 2% of the PTV = 72.76 Gy Maximum dose to 50% of the bladder and rectum = 65 and 50 Gy, respectively Dose to 25% of rectum = 65 Gy
Liver	5 Coplanar fields	45 Gy/3 fx^54^	Dose to 70% of the healthy liver (liver‐CTV) <10 Gy Maximum dose to 30 cm^3^ of heart <30 Gy
Head and neck (H&N)	7 Coplanar fields	Tumor and lymph nodes = 63 Gy/35 fx Gross tumor and its margins = 70 Gy/35 fx^55^, ^56^	Maximum doses of spinal cord = 50 Gy Mean dose of larynx = 40 Gy Mean dose of parotid = 20 Gy^56^, ^57^
Nasal cavity	5 Coplanar fields 2 Non‐coplanar fields	60 Gy/30 fx^58^	Maximum dose of optic nerve/chiasm = 55 Gy Maximum dose of brainstem = 54 Gy Maximum dose of lenses = 20 Gy
GBM	3 Coplanar fields 2 Non‐coplanar fields	60 Gy/30 fx^59^	OAR dose objectives: same as the H&N
Orbits	2 Coplanar fields 6 Non‐coplanar fields	54 Gy/30 fx^60^	OAR dose objectives: same as the H&N

**TABLE 2:** The calculated dose indices for the OARs and PTVs planned by 2.5 and 6 MV photons^a^

		**Prostate**			**Liver**			**H&N**			**Nasal cavity**			**Partial orbit**			**Whole orbit**			**Glioblastoma multiforme**		
		**6 MV (Gy)**	**2.5 MV (Gy)**	**Difference (%)**	**6 MV (Gy)**	**2.5 MV (Gy)**	**Difference (%)**	**6 MV (Gy)**	**2.5 MV (Gy)**	**Difference (%)**	**6 MV (Gy)**	**2.5 MV (Gy)**	**Difference (%)**	**6 MV (Gy)**	**2.5 MV (Gy)**	**Difference (%)**	**6 MV (Gy)**	**2.5 MV (Gy)**	**Difference (%)**	**6 MV (Gy)**	**2.5 MV (Gy)**	**Difference (%)**
PTV	*D*_98%_ (Gy)	66.7	66.5	−0.3	42.1	43.3	2.9	67.2	67.1	−0.1	57.3	57.5	0.2	52.4	52.6	0.4	51.8	52.1	0.6	57.7	57.5	−0.3
	*D*_2%_(Gy)	72.7	72.7	0	45.2	45.4	0.4	72.1	72.1	0.0	61.5	60.2	0.2	54.4	54.8	0.7	55.7	55.5	−0.4	60.2	60.2	0.0
Bladder	*D*_mean_(Gy)	31.4	32.3	2.9																		
	*D*_2%_(Gy)	72	72.1	0.1																		
Rectum	*D*_mean_(Gy)	40.6	41.5	2.2																		
	*D*_2%_ (Gy)	69.8	69.8	0																		
Heart	*D*_mean_ (Gy)				5.7	5.1	−10.5															
	*D*_30 cm3_ (Gy)				15.6	15.5	−0.6															
Liver‐CTV	*D*_mean_ (Gy)				6.4	6.8	6.2															
	*D*_2%_ (Gy)				44.7	44.9	0.4															
Stomach	*D*_mean_ (Gy)				0.7	0.4	*^b^															
	*D*_2%_ (Gy)				5.2	3.1	−40.4															
Spinal cord	*D*_max_ (Gy)				1.1	1.8	63.6	49.8	49.7	−0.2												
Parotid Rt	*D*_mean_ (Gy)							20.3	20.6	1.5												
	*D*_2%_ (Gy)							36.5	35.5	−2.7												
Brainstem	*D*_max_ (Gy)							52.0	52.0	0.0	32.0	29.4	−8.1	5.4	5.0	−7.4	8.9	8.5	−4.5	40.6	39.7	−2.2
Optic Chiasm	*D*_max_ (Gy)										51.1	49.8	−2.5	21.4	19.4	−9.3	36.4	35.2	−3.3	6.3	5.1	−19.0
Optic nerve Rt	*D*_max_ (Gy)										52.4	52.5	0.2							0.5	0.6	*^b^
Optic nerve Lt	*D*_max_ (Gy)										54.9	54.1	−1.5	6.9	7.0	1.4	7.4	7.0	−5.4	0.1	0.9	*^b^
Eye right	*D*_mean_ (Gy)										26.6	26.9	1.1	18.3	19.6	7.1	40.6	45	10.8	0.1	0.2	*^b^
	*D*_2%_ (Gy)										51.3	53.8	4.9	45.9	47.5	3.5	54.3	55.2	1.7	0.2	0.3	*^b^
Eye left	*D*_mean_ (Gy)										25.6	25.8	0.8	0.5	0.5	*^b^	1.6	1.6	0.0	0.1	0.2	*^b^
	*D*_2%_ (Gy)										49.3	52.1	5.7	1.9	3.2	68.4	5.2	5.1	−1.9	0.3	0.4	*^b^
Lens right	*D*_mean_ (Gy)										17.2	15.0	−12.8	8.8	8.3	−5.7	15.5	17.8	14.8	0.1	0.1	*^b^
	*D*_max_ (Gy)										25.0	24.0	−4.0	12.7	12.2	−3.9	22.9	29.9	30.6	0.1	0.1	*^b^
Lens left	*D*_mean_ (Gy)										17.8	15.3	−14.0	0.2	0.3	*^b^	0.5	0.5	*^b^	0.1	0.1	*^b^
	*D*_max_ (Gy)										25.5	22.9	−10.2	0.4	0.5	*^b^	0.7	0.8	14.3	0.1	0.1	*^b^
Brain	*D*_mean_ (Gy)										8.7	7.4	−14.9	2.1	1.8	−14.3	3.7	3.3	−10.8	8.0	7.4	−7.5
	*D*_max_ (Gy)										60.5	59.9	−1.0	53.6	53.6	0.0	54.2	54.9	1.3	60.6	60.7	0.2
Integral dose difference (%)		12.1			11.4			0.7			−7.7			−7.8			−19.2			−6.1		
Average radiological depth (cm)		14.3			14.0			7.5			4.5			5.0			4.5			3.1		
Segment width range (cm)		0.3–7.5			0.3–6.0			0.3–22.5			0.3–8.0			0.3–5.0			0.3–8.0			0.3–6.0		

^a^ For “serial structures”, those tolerance doses are stated in the protocols in terms of max dose, the *D*_max_ is used as the measure for their tolerance doses. Otherwise, the near‐maximum dose, *D*_2%_, is used.

^b^ Regarding the probable large uncertainties for low‐dose values, the absolute doses smaller than 1 Gy have not been taken into consideration in the relative difference calculations.

In the plan optimization, the dose constraints and beam angles were kept the same for both 2.5 and 6 MV plans. Therefore, the only difference was the energy. The matRad TPS takes into account heterogeneities, and uses the photon dose calculation engine PDC++^32^ and employs decomposed pencil beam lateral scattering kernels and depth dose components. The heterogeneity correction is applied based on the radiological pathlength, *d*, of each beamlet according to the Equation (1)^33^:

(1) $$d=\sum_{i}\sum_{j}\sum_{k}l\left(i,j,k\right)\rho \left(i,j,k\right)$$

where ρ(i,j,k) is the density of *(i,j,k)*’th voxel, and *l* is the intersect length of the ray with that voxel.

Although matRad has been developed for research purposes only, the PDC++ is being used in traditional TPSs. The IMRT optimization in matRad is based on influence matrix, in which the delivered dose *d_i_* to voxel *i* is

(2) $$d_{i}=\sum_{j}D_{ij}w_{j}$$

where *D* is the dose contribution matrix in voxel *i* from bixel/beamlet *j*, and *w_j_* is the weight of the bixel. In optimization, depending on the objectives/constraints of each structure and their relative weighting (also called penalty), a cost function is assigned to each individual element. The weighting and constraints were the same for both 2.5 and 6 MV beams. To convert the continuously optimized fluences into deliverable multileaf collimator (MLC) segments, the MLC sequencing algorithm can be enabled. However, as in our study, the practical dose delivery was not considered, this option was not used. The calculation model ignores both the intraleaf transmission and interleaf leakage. Therefore, no MLC parameters are included for both 2.5 and 6 MV beams, except the source to MLC distances.

For prostate and liver, the beamlet (bixel) width was 5 × 5 mm^2^, while a 3 × 3 mm^2^ bixel width was used for the remaining sites. The CORT shared CT images,^34^ and corresponding segmentations were used for planning of the prostate, liver, and H&N cases. The CORT is a shared dataset of CT images, which includes relevant contours for plan comparison in Digital Imaging and Communications in Medicine (DICOM) format. The remaining cases were planned on scans and contouring of anonymized data.

### 2.4 Monte Carlo simulations

Due to the higher photoelectric interaction probability of 2.5 MV beam in bone, an MC simulation was performed to evaluate the accuracy of dose calculation in bone by matRad. In order to compare the absorbed dose in bone and distal to bone between MC and matRad, the MCNPX (version 2.6.0) MC code^35^ was employed. The 2.5 MV photon source with a diameter of 1.0 cm (maximum intensity at the center, and zero in the radius of 0.5 cm) was defined at a distance of 100 cm from the phantom surface (SSD = 100 cm). The calculated PDDs of MC and matRad in a cubic water phantom were matched through tuning of the 2.5 MV spectrum in the MC code for a 10 × 10 cm^2^ field size. The initial spectrum was based on the work of Parsons et al.^17^ Afterwards, the phantom was filled with soft tissue material, and the PDDs with and without the presence of two different bone layers inside the soft tissue phantom were simulated. A bone layer with a density of 1.61 g/cm^3^ and thickness of 0.5 cm at a depth of 1.5 cm (1.5–2 cm) as a representative of cranial bones, and a 4 × 4 × 4 cm^3^ bone block with density of 1.33 g/cm^3^ at the depth of 4 cm (4–8 cm) as a representative of femur bone. The densities and compositions were assigned according to ICRU 46.^36^ Moreover, an MC simulation for a 2 × 2 cm^2^ small field was performed to evaluate the accuracy of matRad calculations for small fields. The photon and electron cutoff were set to 10 and 40 keV, respectively. The mesh‐tally Type 3, which scores the energy deposition from all produced particles, was used to calculate the dose in 1.0 × 1.0 × 0.2 cm^3^ voxels along the central axis, where the 0.2 cm is along the depth direction. The doses to these voxels constitute the points of the PDD curves in the MCNP simulation results. The calculated dose by the mentioned mesh‐tally is a directly calculated dose to material, also referred to as dose to medium‐in‐medium^37^ in the literature. A total of 1.1 × 10^8^ source histories were simulated in order to keep statistical uncertainties below 1.8%.

### 2.5 Clinical evaluations

A nine‐field coplanar plan (gantry angles: 0°, 40°, 110°, 130°, 160°, 200°, 230°, 250°, and 320°) was designed for the prostate case. The liver was planned as stereotactic body radiotherapy (SBRT) composed of five coplanar fields (0°, 170°, 200°, 250°, and 300°).

Simultaneous integrated boost (SIB) technique was used for planning of oropharyngeal H&N cancer. The gantry angles were 0°, 72°, 110°, 144°, 216°, 250°, and 288°. Moreover, the nasal cavity cancer was a localized NK/T‐cell lymphoma. This plan had four coplanar and three non‐coplanar fields with (gantry:couch) angles of 280°:0°, 335°:0°, 0°:0°, 25°:0°, 80°:270°, 325°:270°, and 35°:270°. A GBM tumor case adjacent to brainstem was planned with three coplanar and two non‐coplanar fields (30°:0°, 70°:0°, 110°:0°, 90°:35°, and 90°:325°). Planning for two partial and whole right orbit optic nerve sheath meningioma (ONSM) was performed with two coplanar and six non‐coplanar fields (35°:0°, 300°:0°, 300°:90°, 40°:90°, 35°:45°, 325°:45°, 35°:315°, and 325°:315°).

## 3 RESULTS

### 3.1 Dosimetry data evaluations

#### 3.1.1 Model and configuration of a 2.5 MV photon beam

**FIGURE 1:**
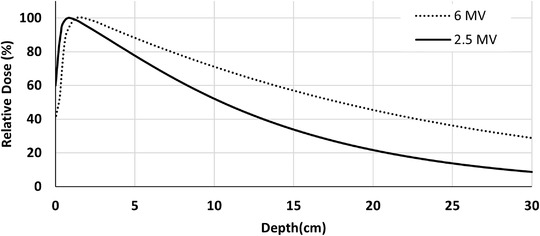
The matRad‐calculated percentage depth dose (PDD) curves of 2.5 and 6 MV photons at SSD = 100 cm and 10 × 10 cm^2^ field size

**FIGURE 2:**
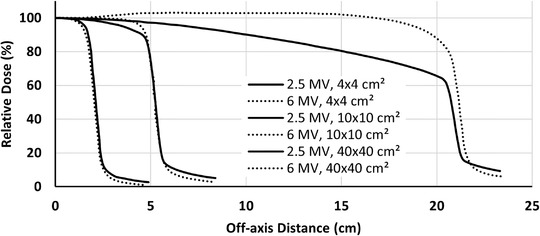
The dose profiles of 2.5 and 6 MV photons at SSD = 100 cm and depth = 5 cm for 4 × 4, 10 × 10, and 40 × 40 cm^2^ field sizes

The comparison between 2.5 and 6 MV PDDs generated by matRad is illustrated in Figure 1. The 2.5 MV beam was commissioned in this work, while the 6 MV beam was a generic model provided in matRad.^29^ The *d*_max_ and PDD at 10‐cm depth (%dd10)^38^ of the 2.5 MV beam were 0.6 cm and 55%, respectively, compared to 1.5 cm and 70% for the 6 MV photons. The dose profiles for the smallest, reference, and largest field sizes (4 × 4, 10 × 10, and 40 × 40 cm^2^) are shown in Figure 2. All dose profiles were normalized to the central axis (CAX) maximum dose value.

**FIGURE 3:**
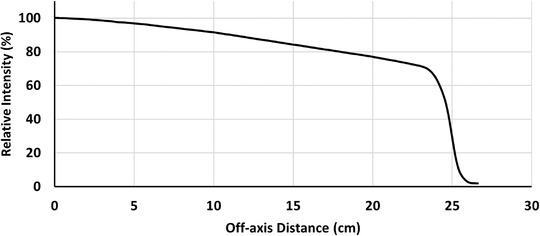
The intensity profile in the diagonal direction at the isocenter used in the matRad data entry that relies on the 40 × 40 cm^2^ dose profile tuning.

**FIGURE 4:**
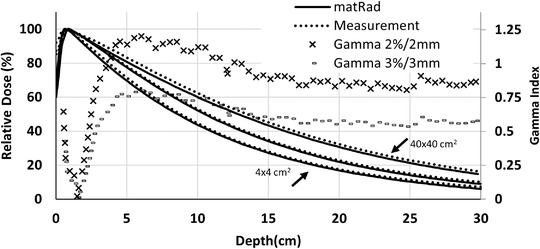
Comparison of the measured and matRad‐calculated percentage depth doses (PDDs) of 4 × 4, 10 × 10, and 40 × 40 cm^2^ field sizes for the commissioned 2.5 MV beam. The presented *γ* indexes are for the PDD of 40 × 40 cm^2^ field size.

Figure 3 shows the tuned intensity profile based on the diagonal dose profile for the largest field at *d*_max_. Subsequently, the calculated PDDs of the selected field sizes (for 4 × 4, 10 × 10, and 40 × 40 cm^2^) are compared with measurements in Figure 4. All PDDs passed the 2%/2 mm *γ* index analysis. However, the PDD of 40 × 40 cm^2^ passed 3%/3 mm *γ* analyses. The maximum deviation for the dose profiles and PDDs were 5% and 3%, respectively, although, in the high‐dose area of the dose profiles (between 100% and 80% isodoses), the difference between the measured and calculated points is under 2%. The maximum 5% deviation of dose profile was for the low‐dose penumbra tail of 40 × 40 cm^2^ field size at the depth of 20 cm.

#### 3.1.2 Monte Carlo simulations

**FIGURE 5:**
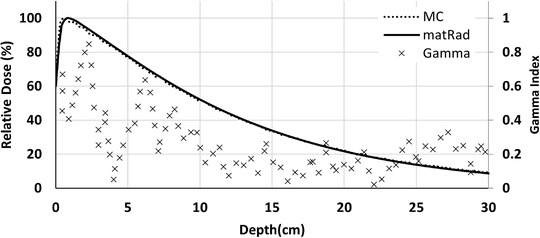
The calculated PDDs of 2.5 MV beam by matRad and MCNP, and corresponding 2%/2 mm *γ* index analysis in the water phantom for 10 × 10 cm^2^ field size at SSD = 100 cm

**FIGURE 6:**
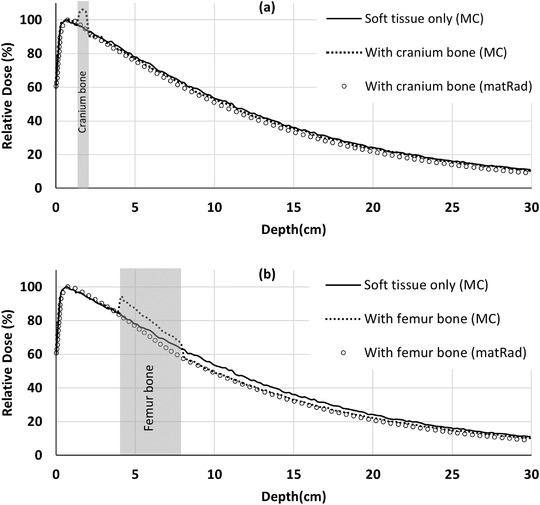
The calculated percentage depth doses (PDDs) in the soft tissue phantom. The dotted lines are the MCNP‐simulated PDDs with the presence of cranium and femur bones. The cranium bone was defined in the depths of 1.5–2 cm, and the femur bone was placed in the depths of 4–8 cm. The solid line is the MC‐simulated PDD without the bones in the soft tissue phantom.

Figure 5 illustrates the calculated PDDs by MC modeling and matRad for 10 × 10 cm^2^ field. The maximum relative difference of the PDDs was 2% for the depths larger than the *d*_max_. However, it was 4% within the buildup region. Subsequently, the PDDs inside the soft tissue phantom were also obtained (Figure 6). The MC simulated PDDs showed, respectively, 10% and 11% dose increment in the cranium and femur bones in comparison to the soft tissue phantom. Besides, the dose to the soft tissue is perturbed after passing through the femoral bone, so that the %dd10 decreased by 3% in comparison to the pure soft tissue phantom. This change is only 1% for the cranial bone. Similar to other pencil beam models, matRad reports dose to “water” not to “material.” Therefore, while the density scaling method in matRad is not capable of modeling dose to bone accurately, it is sufficiently accurate for modeling dose perturbations distal to bone for the purposes of the treatment planning comparisons in this study should a beam pass through bone in a patient case.

**FIGURE 7:**
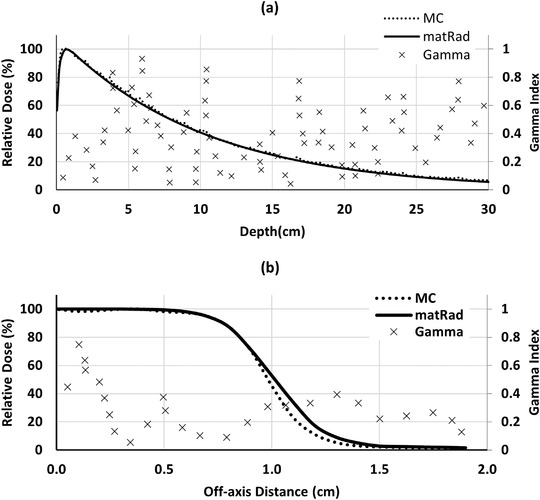
The percentage depth dose (PDD) (a) and dose profile (b) comparison of 2.5 MV beam for a 2 × 2 cm^2^ small field calculated by matRad and MCNP in a water phantom, and corresponding 2%/2 mm *γ* index analysis at SSD = 100 cm. The profile is calculated at the depth of *d*_max_.

As the 2%/2 mm *γ* index analysis shows in Figure 7, the dose distribution for the small field calculated by matRad is in agreement with the MC simulation.

### 3.2 Treatment plan evaluations

**FIGURE 8:**
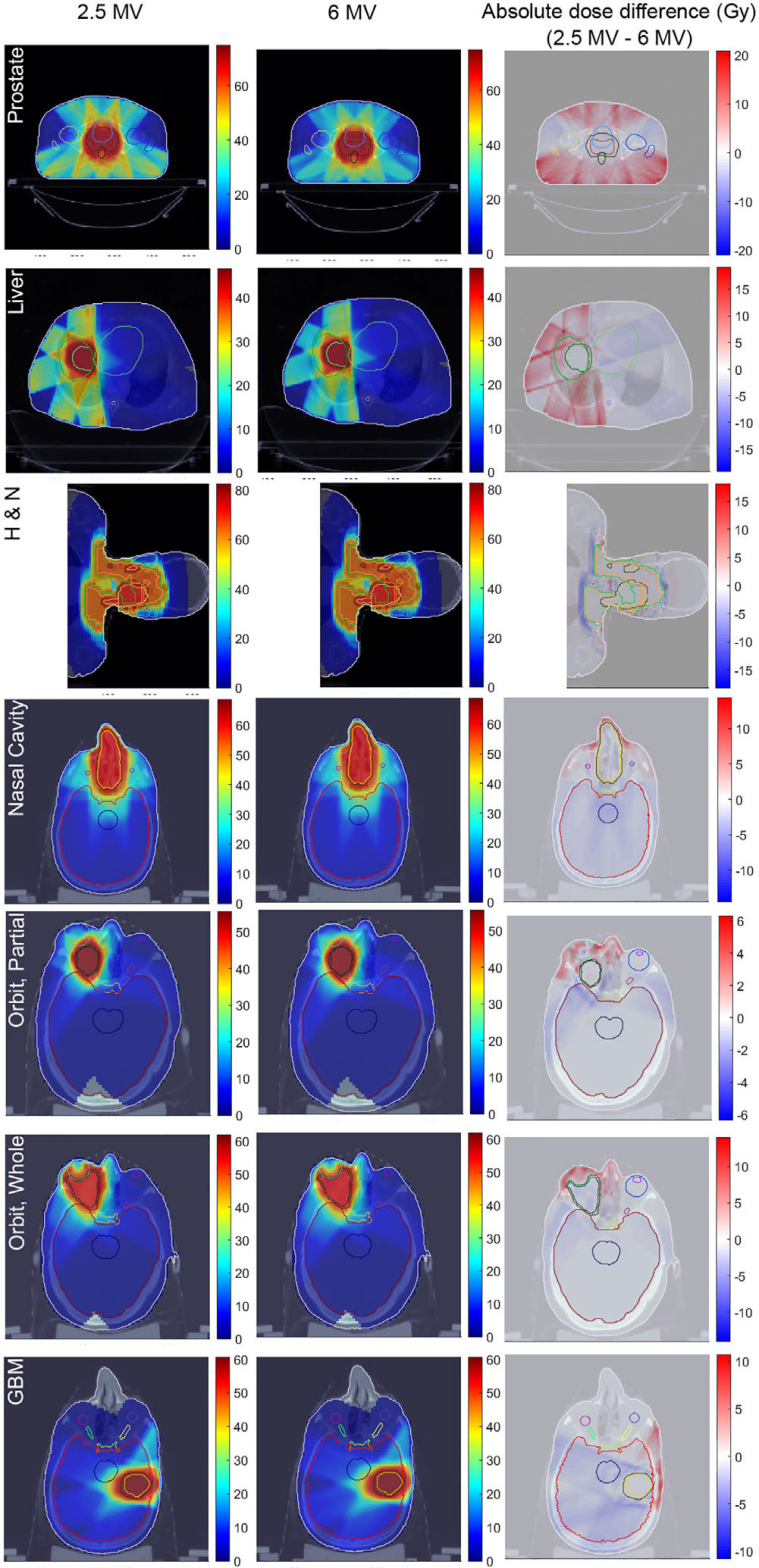
The dose distribution of 2.5 and 6 MV photons plans, as well as their absolute difference and *γ* index comparisons.

As shown in Table 2, in terms of PTV coverage, the two beams are almost the same, so that the relative difference between *D*_98%_ (dose to 98% of the volume) of prostate PTV for both beams is 0.3%. However, the mean doses of rectum and bladder by the 2.5 MV beam were 2.2% and 2.9% higher than 6 MV photons. In contrast, the near‐maximum doses, *D*_2%_ (dose to 2% of the volume), of the rectum and bladder were equal. Despite the acceptable dose to OARs for both beams, the 2.5 MV plan delivers 12.1% higher integral dose (sum of the doses to all voxels divided by total volume) to the prostate patient. The dose distributions and absolute dose difference are illustrated in Figure 8.

In the liver plan, the mean dose to the heart from 2.5 MV beam is 10.5% lower than 6 MV beam. However, the integral dose and spinal cord dose of 2.5 MV photons was 11.4% and 63.6% higher than 6 MV photons, respectively. In the H&N plan, the dose differences of OARs and targets from two beams were under 3%. All other considered structures had similar DVHs for both beams. The integral doses of two beams were also almost equal.

**FIGURE 9:**
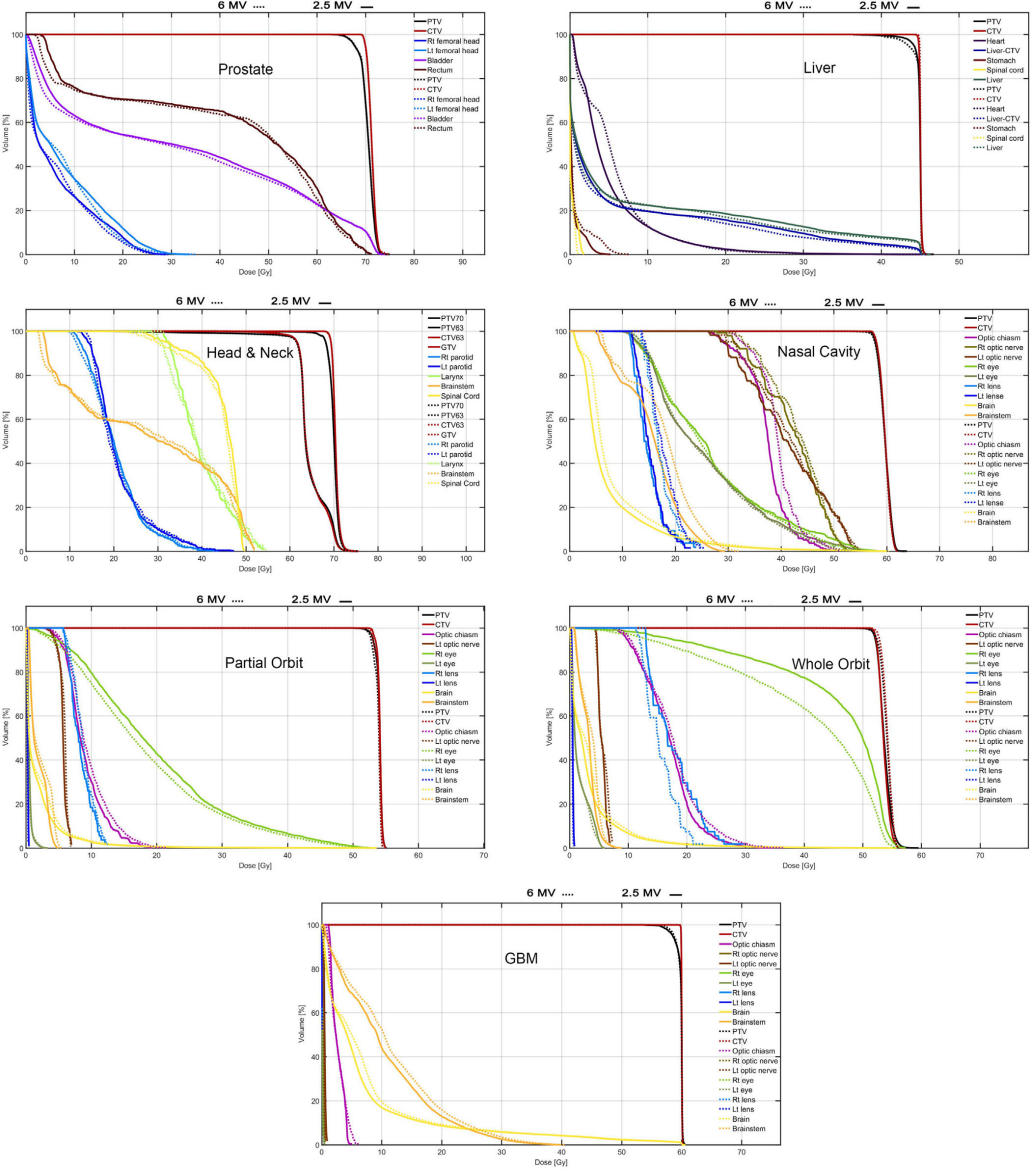
Dose‐volume histograms (DVH) comparison between 6 and 2.5 MV beams for different cases

As it can be seen in Figure 9 and Table 2, the mean, max, and *D*_2%_ doses of OARs (except the eyes) of the nasal case planned by the 2.5 MV photon receive less than or equal to the 6 MV plan. For example, the max dose of brainstem and the mean doses of eye lenses were 8.1% and 12.8% lower than 6 MV beam. In addition, the integral dose of 2.5 MV photon was 7.7% less than 6 MV photons.

According to Table 2 and Figure 9, the orbit DVHs demonstrated that the 2.5 MV beam delivers less doses to the OARs, apart from the right lens and eye in the whole orbit, and the right eye in the partial orbit plans. Furthermore, the integral doses of the 2.5 MV plans on the partial and whole orbit were 7.8% and 19.2% lower than the plans of 6 MV beam.

The GBM case integral dose was 6.1% lower for the 2.5 MV plan. In terms of the doses to the normal tissues and PTV coverage, both plans were very similar, so that the dose differences between PTV coverage, brain, and brainstem maximum doses for both situations were within 1%. On the other hand, the 2.5 MV plan showed 19.0% lower maximum dose to the optic chiasm, and 7.5% lower mean dose in the brain.

## 4 DISCUSSION

The comparison between the calculated and measured PDDs of the 2.5 MV beam showed a maximum deviation of 3% at the largest field size. This may possibly be because the measured PDDs when converted to TMRs, did not include PSF data, as it was assumed to be unity. This means that the calculations underestimate the PDDs up to 3% for large field sizes.

While comparing the plans with the two energy beams, all the OARs and PTVs passed the desired dose constraints and objectives for both beams. In addition, the difference in *D*_98%_ between the 2.5 and 6 MV, an indicator for evaluating the near‐minimum dose to a structure, for the PTVs was under 2%, except for the liver, which was 2.9%. Similarly, the *D*_2%_ differences, near‐maximum dose index, were less than 1%. However, the situation of the OARs varied case by case. As it can be seen in the absolute dose differences in Figure 8, the liver, nasal cavity, orbit, and GBM plans in which the majority of their fields are arranged ipsilaterally, the 6 MV beam delivers lower doses to the areas before the PTVs and higher doses to the organs beyond the PTVs. This is due to the deeper *d*_max_, and slower PDD fall‐off of 6 MV relative to 2.5 MV beam. Given that the heart mean dose and stomach maximum dose in the liver plan, brainstem, and optic chiasm maximum doses, brain mean dose, and the integral doses in the nasal cavity, orbit, and GBM plans were lower for the 2.5 MV beam. The lower dose to the deep‐seated organs such as stomach and heart doses in the liver case, plus lower integral doses would be advantageous in re‐irradiation scenarios and reducing the risk of secondary cancers. In contrast, the integral dose of the plans with deep‐seated tumors, namely, prostate and liver, were higher for the 2.5 MV, due to its higher dose gradient.

Achieving a rapid dose fall‐off beyond the target (PTV), and consequently, reducing the dose of adjacent OARs using low MV has been traditionally used in radiotherapy in general and especially in LINAC‐based stereotactic radiosurgery (SRS).^39^, ^40^ By designing hybrid plans with higher energies, the steeper fall‐off of PDD with 2.5 MV photons can be used in treatment of cases where normal tissues with low‐dose tolerances, such as the heart in the liver case, are in the path of the beam exit. For example, in the liver plan, the higher energy fields can be arranged in the anterior and posterior directions that do not pass through the heart, but the lateral fields, which exit through the heart could be planned with 2.5 MV fields. Unfortunately, the current version of matRad only allows selection of single energy for a given plan, and multiple beam energy optimization is beyond the scope of this current work. The combination of multiple energy beams in a single plan has been studied previously.^10^

In a study by Zhang et al.,^21^ treatment planning with a 3 MV photon beam for 31 lung cancer patients was investigated. They reported a better target coverage by the 3 MV beam in comparison to a 6 MV beam. For short radiological depth cases (<10 cm), the dose to OARs were reduced between 7.4% and 11.4%. In addition, the integral doses were lower than the 6 MV beam. Similarly, another study^23^ showed that the dose to OARs of H&N, liver, and breast in the SBRT plannings is reduced by a 2 MV photon compared with a 6 MV beam. These findings are coincident with our results for the cases with short average radiological depths in Table 2, as discussed above.

The 2.5 MV beam has a peak and mean energies of around 120 and 500 keV, respectively,^17^, ^19^ compared to 1 and 1.5 MeV,^41^ respectively, for a 6 MV beam. The considerable amount of low‐energy photons in the 2.5 MV beam can lead to a dose enhancement if used in combination with gold nanoparticles (GNPs). An MC study by Tsiamas et al.^28^ investigated the microscopic dose enhancement ratio (DER) caused by 2.5 MV beam with low‐*Z* targets. The DER for a low‐Z 2.5 MV beam was approximately two times higher than a conventional high‐*Z* 4 or 6.5 MV beam. They also compared the photon energy spectrums and PDDs of different thicknesses diamond (high‐density carbon) and beryllium (Be) targets. At a depth of 2 cm, the DER was almost the same for different targets and different materials of 2.5 MV beam. However, at a depth of 22 cm, DER for the Be target was around 25% higher than diamond target. A recent study demonstrated that 10 μg/ml of GNPs can cause dose enhancement of 2.5 MV beam compared to 6 MV photons.^1^ The results of this current study indicate that the 2.5 MV beam can produce clinically acceptable treatment plans. Future work will investigate the combined effect of 2.5 MV beam and GNPs to optimize the therapeutic ratio for different treatment sites.

According to the MC simulations, matRad underestimated the bone doses, which is due to the inability of regular computed tomography (CT)‐based TPSs to calculate photoelectric effect dose. Conventional TPSs use CT‐to‐density table in their dose calculations in different voxels where no information on tissue *Z* is available. Furthermore, the Compton interaction is dominant for conventional treatment energies of 6–18 MV. As the Compton interaction is dependent on the electron density and is largely independent of *Z*, while the photoelectric effect is strongly related to *Z*, conventional TPSs correct for inhomogeneities impacting Compton interactions and score the energy mainly released by Compton not photoelectric effect. On the other hand, Compton scattering has a mild relation with the photon energy; however, the photoelectric effect is much more likely for energies in the keV region. Therefore, regarding the higher *Z* of bone elements in comparison to soft tissue materials, as well as the low‐energy spectrum of the 2.5 MV beam, the photoelectric effect can lead to inaccuracy of dose calculations in dense and thick bones, like femoral heads, as observed in Figure 6. In addition, for thick bones, the depth dose values decreased after passing through the bone. Hence, for the prostate case, the fields were arranged in such a way that they did not pass through the femurs in order to avoid these additional uncertainties. Although the AAPM TG‐180 report^24^ has not mentioned the increase in the dose to the bones by 2.5 MV energy, it points to the application of developing low‐energy dose kernels in compensating for the underestimation of the dose to bone in commercial TPS for kV imaging photons. Their dose estimation was done through taking into consideration of the bone density instead of *Z* and corresponding photoelectric effect.^14^ Similarly, new kernels are needed to be defined in future studies to estimate the bone doses by 2.5 MV photons to answer this question and evaluate the accuracy of PDC++ algorithm in calculating the dose distribution of this photon beam.

Apart from the indicated physical aspects, the 2.5 MV beam has *d*_max_ and %dd10 values similar to cobalt‐60 (^60^Co) teletherapy machines,^42^ which have been used for many years in developing and low‐income countries due to their low cost and low maintenance. However, they have almost been discontinued due to some drawbacks such as large geometrical penumbra, short half‐life, and consequently the need to replace the source every few years, in addition to the safety and security concerns of radioisotope sources. The production of 2.5 MeV electrons, and consequently 2.5 MV photons, requires 1–2 MW magnetrons that are more economical in terms of power consumption, replacement costs, and related parts such as modulator and cooling system.^43^, ^44^, ^45^ The magnetron (or klystron) power for conventional LINACs are above 5 MW, that of course has longer accelerator‐waveguides as well.^46^, ^47^ Therefore, a compact LINAC with an energy of 2.5 MV beam can be a replacement for the ^60^Co machines and an alternative for conventional LINACs in those countries. Thus, providing a treatment unit with IMRT capabilities that is more cost‐effective than traditional ^60^Co treatments.^48^, ^49^ Such a machine, due to the lower energy and tenth‐value layer (TVL) in comparison to 6 MV photons,^50^, ^51^ requires less LINAC head and room shielding, thinner MLC and jaws, and smaller footprint.

Practical considerations such as dose rate for beam generation were beyond the scope of this work. As the 2.5 MV photon beam on TrueBeam LINAC is commercially designed for imaging, its dose rate has been set to 60 MU/min^50^, ^51^ by default, while typical dose rates of a therapeutic 6 MV beam are in the range of 200–1400 MU/min. This low‐dose rate can be addressed by increasing the electron gun current, and re‐designing of the target cooling system if necessary. The output energy of a LINAC is independent from the dose rate (pulse reputation frequency, PRF) of injected electrons, but depends only on the current of bending magnet and RF pulse height.

This work compared the physical properties and treatment planning of 2.5 MV beam with the 6 MV photon. The MC simulations versus pencil beam algorithm comparisons demonstrated the need for investigations on development of low‐MV‐specific dose kernels to calculate the dose to bone accurately. Therein, the dose calculations must be done based on low‐energy kernels and attenuation coefficient data rather than electron densities.

This work is not aimed to provide direct clinical use and commissioning of the 2.5 MV beam, but to evaluate the feasibility of treatment planning and its capabilities in treatment. For this reason, in the future works, TPS with a more accurate dose computation algorithm than pencil beam, as well as a model based on measurements with smaller detectors should be considered. Therein, following the clinical commissioning guidelines such as MPPG 5.a^52^ will be necessary.

## 5 CONCLUSION

A novel 2.5 MV beam was configured in a research system to investigate the treatment plan possibilities of the imaging beam. The PTV dose coverage metrics (*D*_2%_ and *D*_98%_) for all investigated cases did not show a difference larger than 3% between 2.5 and 6 MV beams. For shallow targets such as orbit, nasal cavity, and GBM the 2.5 MV photons, and for deeper targets like prostate and liver cancers, the 6 MV beam presented lower integral doses. In addition, most OARs in the nasal cavity and GBM cases were better spared by the 2.5 MV beam. Due to the lower exit dose of 2.5 MV beam, the distal OARs received lower mean doses in comparison with the 6 MV beam. This can be considerable gain in the re‐irradiation of some sites in case of tumor recurrence or metastases.

The study also alludes to the fact that the 2.5 MV beam can be considered in the design of single‐energy LINACs, where the low cost, reduced power consumption, minimal operating costs are critically important such as in low‐ and middle‐income countries. In addition, this comparison of these two model beams demonstrates the potential for treatment planning with the 2.5 MV beam with the potential added benefit of dose enhancement if used in combination with high‐*Z* nanoparticles.

## AUTHOR CONTRIBUTIONS

All the authors contributed directly to the intellectual content of the paper. They meet all of the criteria indicated on “Authorship” section of JACMP website.

## CONFLICT OF INTEREST

The authors have no relevant conflicts of interest to disclose.
